# Assessment of Sexual Function in Relation to Microbiological Findings in Patients with Chronic Bacterial Prostatitis

**DOI:** 10.3390/diagnostics15070887

**Published:** 2025-04-01

**Authors:** Vittorio Magri, Gianpaolo Perletti, Konstantinos Stamatiou, Alberto Trinchieri

**Affiliations:** 1 Urology Clinic, ASST Fatebenefratelli Sacco Hospitals, 20026 Milan, Italy; vmdoctor26@gmail.com; 2 Department of Biotechnology and Life Sciences, Section of Medical and Surgical Sciences, University of Insubria, 21100 Varese, Italy; gianpaolo.perletti@uninsubria.it; 3 Urology Department, Tzaneio General Hospital, 18536 Piraeus, Greece; stamatiouk@gmail.com; 4 CDC Ambrosiana, Cesano B, 20090 Milan, Italy

**Keywords:** bacterial chronic prostatitis, erectile dysfunction, premature ejaculation, ejaculatory pain, sexual dysfunction

## Abstract

**Background/Objectives**: Patients with chronic bacterial prostatitis (CBP) often present symptoms of sexual dysfunction. We aimed to evaluate the impact of the infection location and etiology on sexual dysfunction in patients with CBP. **Methods**: Male patients with CBP diagnosed by microbiological tests underwent a complete clinical assessment and were administered questionnaires for prostatitis (NIH-CPSI), voiding (IPSS), and sexual function (IIEF-15, PEDT). **Results**: Out of 614 patients, erectile dysfunction (ED) was present in 49.8%, and premature ejaculation (PE) in 40.7%. At least one sexual disorder was present in 86.3% when other disorders of ejaculation, orgasm, and sexual desire were considered. Patients with Gram-negative infections in expressed prostatic secretion (EPS) or voided urine after prostatic massage (VB3) had higher odds of moderate to severe erectile dysfunction compared to patients with infection by atypical pathogens (OR 3.31, CI 1.43–7.63, *p* = 0.0039). Rates of orgasmic dysfunction were also higher in Gram-negative and Gram-positive with respect to atypicals (OR 3.2, CI 1.36–7.90, *p* = 0.006 and OR 3.78, CI 1.64–8.71, *p* = 0.001). Hemospermia was more frequent in patients with semen infection by Gram-positive than in patients with infection by atypical pathogens (OR 2.2984, CI 1.3239–3.9901, *p* = 0.002). Prostatic calcifications at transrectal ultrasound were less frequent in patients with semen infection by Gram-negative compared to Gram-positive (OR 0.471, CI 0.3029–0.7322, *p* = 0.000). The addition of an “S” (sexual) domain to the UPOINT classification achieves a more significant correlation between the number of positive domains in each patient and the NIH-CPSI score. **Conclusions**: Infections by Gram-negative are associated with more sexual morbidity in patients with CBP. The use of a questionnaire investigating all the main domains of sexual dysfunction could be very useful for the phenotyping of patients with chronic prostatitis.

## 1. Introduction

About 20 years ago, the frequent association of symptoms of sexual dysfunction in patients with chronic prostatitis/chronic pelvic pain syndrome (CP/CPPS) was described [1]. Patients with CP/CPPS are diagnosed based on the results of microbiological tests [2] demonstrating bacteria in prostate secretion, in urine voided after prostate massage, or in seminal fluid. Patients in whom the presence of bacteria has been demonstrated are diagnosed as chronic bacterial prostatitis (CBP) or category II, those in whom the presence of bacteria has not been demonstrated employing standard microbiological techniques but have chronic pelvic pain are classified as category III, which can, in turn, be divided into inflammatory (IIIa) or non-inflammatory (IIIb) based on the presence of leukocytes in the prostate-specific samples examined [3].

Rates of erectile dysfunction (ED) and semen abnormalities were reported to be significantly higher in patients with bacterial chronic prostatitis compared to patients with chronic pelvic pain syndrome [1].

Several studies have since confirmed the frequent association between sexual dysfunction and CP/CPPS. A meta-analysis [4] of three cross-sectional studies, two case-control studies, and four retrospective studies demonstrated a strong correlation between CP/CPPS and ED (pooled odds ratio: 3.02). Men with a self-reported diagnosis of CP/CPPS reported mild to severe erectile dysfunction in 47.4% of cases [5]. Another meta-analysis [6], which included 24 studies involving 11,189 men, demonstrated an overall prevalence of sexual dysfunction in men with CP/CPPS of 62%, with a prevalence of erectile dysfunction and premature ejaculation of 29% and 40%, respectively. 

Some authors [5,7,8,9] emphasize the importance of penile, testicular, ejaculatory, and/or perineal pain symptoms, which interplay with cognitive and emotional factors in inducing alterations of sexual functioning in these men with CP/CPPS.

Other authors have instead considered sexual dysfunction as secondary to pathophysiological alterations related to the inflammation of the genital tract, identifying it as one of the phenotypic domains of CP/CPPS syndrome. For this reason, it has been proposed to add a sexuality-specific domain in the UPOINT (urinary, psychosocial, organ-specific, infection, neurologic/systemic, tenderness) [10] phenotypic classification system, although conflicting opinions still exist on this [11].

The aim of this study was to evaluate the association between sexual dysfunction and the characteristics of genital infection in terms of biological samples examined for diagnosis and type of pathogens. A secondary aim of the study was to highlight the results of a more extensive assessment of sexual function in patients with CBP beyond erectile function alone but including all other domains of male sexual function.

## 2. Materials and Methods

This cohort study was based on the retrospective analysis of a database of patients who were consecutively observed at an outpatient prostatitis clinic over a 20-year period from January 2004 to June 2024.

Inclusion criteria: Patients who were male, aged 18 to 65 years, with symptoms or signs suggestive of chronic prostatitis according to the NIH-CPSI questionnaire [3]. Patients were included based on the detection of pathogens in urine after prostatic massage (VB3) or in expressed prostatic secretion (EPS) during a Meares–Stamey test [2] or in seminal fluid.

Exclusion criteria: Patients with urethral discharge or symptoms suggestive of urethritis and patients with symptoms of acute prostatitis, including acute urinary symptoms, urinary retention, fever, or general malaise, were excluded.

Patients were also excluded if they had indwelling catheters, previous prostatic surgery or radiotherapy, neoplasia, or any serious disease or condition that might represent a major confounder in the evaluation of the patients.

A total of 614 patients were studied with medical history (age, previous history of urinary tract infection or sexually transmitted infection, premature ejaculation, hemospermia, vascular risk factors for erectile dysfunction), microscopic and microbiological tests (Meares–Stamey test and sperm culture) [2], prostate-specific antigen (PSA) measurement, uroflowmetry (Qmax, post voiding residual), prostatic transrectal ultrasound for evaluation of prostatic fibro-calcifications; symptomatic questionnaires such as the National Institute of Health-Chronic Prostatitis Symptom Index (NIH-CPSI) [3], the International Prostate Symptom Score (IPSS) [12], the International Index of Erectile Function (IIEF-15) [13], and the Premature Ejaculation Diagnostic Tool (PEDT) [14], UPOINT [10].

### Statistics

Statistical analyses were conducted using the “R” (v4.3.3) environment for data analysis and statistical computation. Continuous variables were reported as mean values and standard deviations. Categorical variables were reported as the number of cases (n°) and percentage (%), and their differences were correlated with the Pearson x^2^. Pearson’s correlation coefficients were calculated to analyze correlations between normally distributed variables. Odds ratios and 95% confidence intervals were calculated.

Multivariable logistic regression models were used to assess associations of age, clinical variables, and questionnaire scores with erectile dysfunction, orgasmic dysfunction, sexual desire, and premature ejaculation.

Spearman correlation analysis was used to study the correlation between the number of positive domains of the UPOINT(S) system and NIH-CPSI and IIEF-15 scores. Comparisons were considered to differ significantly if *p* < 0.05.

## 3. Results

### 3.1. Microbiological Findings

Post-massage urine VB3 was positive in 202 of 614 samples, semen culture in 448 of 614 samples, and prostatic secretion in 101 of 129 of the samples that could be collected after digital prostatic massage.

In 141 patients, pathogens were present in both the Meares–Stamey test [2] samples (EPS and/or VB3) and in the sperm culture, in 155 cases only in the Meares–Stamey test and in 307 cases only in the semen. Of the 296 cases with positive Meares–Stamey, 195 had only positive VB3, 94 only positive EPS, and 7 were both positive.

**Figure 1:**
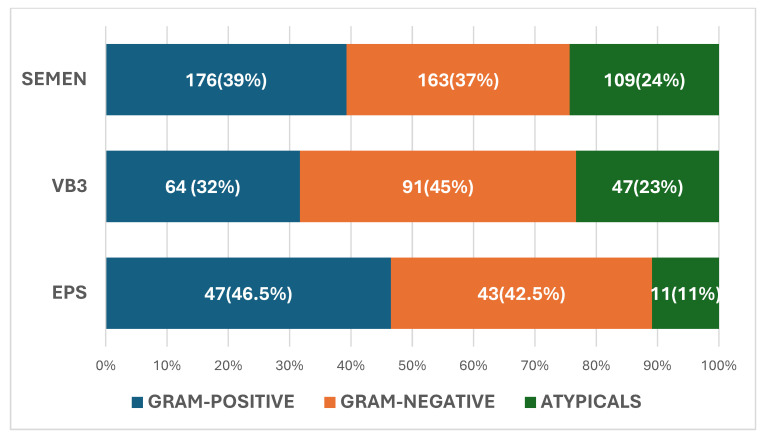
Spectrum of pathogens in post-massage urine VB3 expressed prostatic secretion (EPS) and seminal fluid.

The pathogens isolated in VB3, EPS, and seminal fluid are described in Figure 1. In VB3, 91 were Gram-negative (45%), 64 were Gram-positive (32%), and 47 were atypical pathogens (23%) (total 202). In seminal fluid, 163 were Gram-negative (37%), 176 were Gram-positive (39%), and were 109 atypical (24%) (total 448). In EPS, 43 were Gram-negative (42.5%), 47 were Gram-positive (46.5%), and 11 were atypical (11%) (total 101).

#### 3.1.1. VB3 Urine

In urine VB3 we isolated 91 Gram-negative strains (*Escherichia coli*, 52, *Proteus mirabiis* 11, *Pseudomonas aeruginosa* 4, *Klebsiella* spp. 6, *Enterobacter* spp. 2, *Citrobacter* spp. 3, *Morganella morganii* 4, *Haemophilus parainfluenzae* 8, and *Neisseria* spp. 1), 64 were Gram-positive (*Enterococcus* spp. 23, coagulase-negative staphylococci or CoNS 4, *Staphylococcus aureus* 6, *Staphylococcus epidermidis* 3, β-hemolytic streptococci or BHS 14, *Streptococcus agalactiae* 4, *Streptococcus uberis* 1, *Streptococcus bovis* 1, *Peptostreptococcus* spp. 1, *Kocuria kristinae* 2, *Corynebacterium* spp. 6) and 47 atypicals (*Chlamydia trachomatis* 21, and *Ureaplasma urealyticum* 26).

#### 3.1.2. Semen

In semen we isolated 163 Gram-negative strains (*E. coli* 93, *Proteus mirabiis* 12, *Pseudomonas aeruginosa* 5, *Klebsiella* spp. 9, *Enterobacter* spp. 1, *Citrobacter* spp. 10, *Morganella morganii* 11, *Haemophilus parainfluenzae* 19, *Acinetobacter* spp. 1, and *Neisseria* spp. 2), 176 Gram-positive (*Enterococcus* spp. 98, coagulase-negative staphylococci or CoNS 5, *Staphylococcus haemolyticus* 6, *Staphylococcus aureus* 3, *Staphylococcus epidermidis* 2, *Staphylococcus anginosus* 6, *Staphylococcus hominis* 2, *Staphylococcus lugdunensis* 1, *Staphylococcus saphrophiticus* 1, β-hemolytic streptococci or BHS 23, *Streptococcus agalactiae* 7, *Streptococcus mitis* 5, *Streptococcus uberis* 1, *Streptococcus bovis* 1, *Streptococcus viridans* 4, *Peptostreptococcus* spp. 1, *Kocuria kristinae* 3, *Corynebacterium* spp. 5), *Gardnerella vaginalis* 2 and 109 atypicals (*Chlamydia trachomatis* 21, *Ureaplasma urealyticum* 85, and *Mycoplasma hominis* 3).

#### 3.1.3. Expressed Prostate Secretion (EPS)

In EPS were isolated 43 Gram-negative strains (*E. coli* 38, *Proteus mirabilis* 2, *Klebsiella* spp. 1, *Enterobacter* spp. 1, and *Haemophilus parainfluenzae* 1), 47 Gram-positive (*Enterococcus* spp. 20, coagulase-negative staphylococci (CoNS) 5, *Staphylococcus haemolyticus* 4, *Staphylococcus aureus* 3, *Staphylococcus epidermidis* 1, β-hemolytic streptococci or BHS 8, *Streptococcus agalactiae* 3, *Streptococcus mitis* 1, *Corynebacterium* spp. 2) and 11 atypicals (*Chlamydia trachomatis* 6, and *Ureaplasma urealyticum* 5).

#### 3.1.4. Identical Pathogens in Different Biological Samples

The same pathogen was isolated at the same time in EPS and VB3 urine in only 7 cases (*E. coli* 2, *Proteus mirabilis* 1, *Enterococcus faecalis* 1, Coagulase-negative staphylococcus 1, and *Ureaplama urealyticum* 2), in EPS and semen in 29 cases (*E. coli* 8, *Proteus* spp. 2, *Pseudomonas* spp. 1, *Haemophylus parainfuenzae* 1, *Enterococcus faecalis* 6, β-hemolytic streptococci (BHS) 4, *Streptococcus mitis* 1, α-hemolytic streptococci 1, coagulase-negative staphylococcus (CoNS) 1, *Staphylococcus haemolyticus* 1, *Chlamydia trachomatis* 2, and *Ureaplasma urealyticum* 1) and in VB3 urine and semen in 79 cases (*E. coli* 15, *Proteus mirabilis* 4, *Psudomonas aeruginosa* 1, *Klebsiella pneumoniae* 2, *Klebsiella oxytoca* 1, *Citrobacter koseri* 2, *Haemophylus parainfuenzae* 3, *Morganella morganii* 1, *Enterococcus faecalis* 12, *Enterococcus faecium* 2, *Steptococcus uberis* 1, *Streptococcus agalactiae* 1, β-hemolytic streptococci (BHS) 5, *Peptostreptococcus* spp. 1, *Kocuria kristinae* 2, *Staphylococcus epidermidis* 1, *Staphylococcus aureus* 1, *Corynebacterium* spp. 2, *Chlamydia trachomatis* 9, and *Ureaplasma urealyticum* 13). The same pathogen was isolated at the same time in EPS, urine VB3, and semen in only one case (*Enterococcus faecalis*).

### 3.2. Demographics and Clinical Characteristics

Table 1 shows the demographic data of the studied population, mean values ± standard deviations of some objective parameters related to chronic prostatitis (white blood count in urine after prostate massage and in seminal fluid, PSA, maximum flow, and post-voiding bladder residual urine), mean values ± standard deviations of the scores of some questionnaires related to the severity of symptoms related to prostatitis (NIH-CPSI), urinary disorders (IPSS), sexual function (IIEF-15, PEDT) and the presence (as numbers and percentages) of some pathological conditions related to chronic prostatitis (prostatic calcifications, hemospermia, premature ejaculation, and presence of metabolic risk factors for erectile dysfunction).

Multiple regression was performed to analyze factors explaining erectile dysfunction, orgasmic dysfunction, loss of desire, and premature ejaculation. Independent variables included age, PSA value, uroflowmetry parameters, white blood count in VB3 urine and semen, scores of NIH-CPSI and IPSS questionnaires, and vascular risk factors.

Multiple regression analysis identified age (β = −0.113, *p*  =  0.030), PSA (β = −0.10, *p* = 0.047), and vascular risk factors (β = −0.120, *p*  =  0.007) as independent predictors of erectile dysfunction (adjusted R^2^ = 0.07). Age (β = −0.142, *p*  =  0.006) and vascular risk factors (β = −0.091, *p* = 0.040) were independent predictors of orgasmic dysfunction (adjusted R^2^ = 0.084). PSA (β = 0.109, *p* = 0.032) and vascular risk factors (β = −0.120, *p* = 0.008) were independent predictors of loss of desire (adjusted R^2^ = 0.065). No significant predictors of premature ejaculation were identified.

The correlations for erectile dysfunction are negative because IIEF scores tend to be lower with worsening sexual function.

### 3.3. Biological Samples and Type of Infection

The impact of the biological material where pathogens were isolated (VB3, EPS, semen) and the type of pathogens (Gram-negative, Gram-positive, and atypical) on IIEF-15 domains for erectile function, orgasmic function and sexual desire and on the presence of premature ejaculation, hemospermia, and prostatic calcifications were presented in Table 2. No significant difference was observed in relation to the source of the biological sample. Patients with infection of EPS/VB3 urine by Gram-negative strains showed a higher rate of moderate to severe erectile dysfunction than patients with atypical pathogens (OR 3.3115, CI 1.435–7.642, *p* = 0.0039). Rates of orgasmic dysfunction were higher in Gram-negative and Gram-positive with respect to atypicals (OR 3.284, CI 1.3641–7.9058, *p* = 0.006 and OR 3.7824, CI 1.641–8.7182, *p* = 0.001). Hemospermia was more frequent in patients with semen infection by Gram-positive than in patients with infection by atypical pathogens (OR 2.2984, CI 1.3239–3.9901, *p* = 0.002.). Prostatic calcifications at transrectal ultrasound were less frequent in patients with semen infection by Gram-negative compared to Gram-positive (OR 0.471, CI 0.3029–0.7322, *p* = 0.000).

### 3.4. Prevalence of Sexual Dysfunction Symptoms

The IIEF 1–5,15 score demonstrated an alteration of erectile function (sum of the answers to questions 1–5 plus the answer to question 15) in 306 patients (49.8%), which was rated as severe in 28 cases (4.8%), moderate in 70 (11.4%), mild in 208 (33.9%). Erectile dysfunction was absent in 308 (50.2%).

The reply to an unstructured interview demonstrated a rate of premature ejaculation of 40.7% (250/614). The use of the PEDT questionnaire in a subsample of 111 patients found the absence of premature ejaculation (PE) in 60.4%, probable PE in 8.1%, and the presence of PE in 31.5%. The presence of orgasmic dysfunction (IIEF 9 and/or IIEF 10 ≤ 2) was observed in 173 (28.1%), the presence of impairment of sexual desire (IIEF 11 and/or IIEF 12 ≤ 2) in 170 (27.6%) and positive reply to question NIH-CPSI on ejaculatory pain in 375 (61.0%).

**Figure 2:**
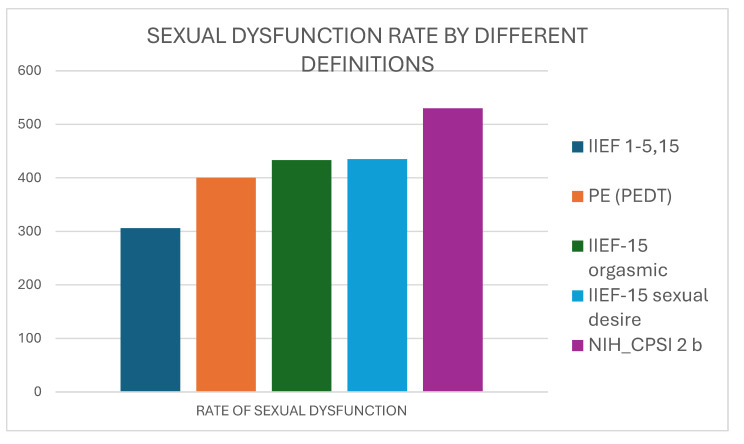
The increasing rate of sexual dysfunction by considering IIEF score for erectile dysfunction (IIEF 1–5,15) alone or together with premature ejaculation rate, IIEF-15 scores for orgasmic function or desire, and NIH-CPSI reply to question 2b for ejaculatory pain.

Figure 2 shows that the rate of sexual dysfunction calculated by the results of the IIEF-15 erectile function score progressively increased up to 86.3% when premature ejaculation, orgasmic dysfunction, desire impairment, and ejaculatory pain were considered.

### 3.5. UPOINT Classification

Patients were divided according to UPOINT classification. They were assigned to the urinary domain “U” (318, 51.8%), psychologic domain “P” (174, 28.3%), organ-specific O (467, 76%), neurologic domain (252, 41%), and tenderness domain (321, 52.2%). Obviously, all patients were positive for domain I (infection). The median number of positive domains was 4.

The number of positive UPOINT domains was significantly correlated with total NIH-CPSI score (0.262, *p* = 0.000), pain NIH-CPSI score (0.164, *p* = 0.000), micturition NIH-CPSI (0.272, *p* = 0.000) and QoL NIH-CPSI (0.245, *p* = 0.000). On the contrary, the number of positive domains was not significantly correlated to any of the scores of IIEF-15 (erectile, orgasmic, and sexual desire) (Table 3).

### 3.6. UPOINTS

We initially calculated the value of a “S” domain for sexual dysfunction using the rate of erectile dysfunction calculated by IIEF 1–5,15 erectile score, and subsequently, we calculated it considering the presence of other sexual dysfunctions, including premature ejaculation, orgasmic dysfunction, impairment of sexual desire and ejaculatory pain.

When we used the erectile domain of the IIEF-15 score to assign positivity to “S” domain, we included 306 patients (49.8%) with mild to severe erectile dysfunction. The use of the erectile IIEF-15 domain in combination with the rate of premature ejaculation (according to an unstructured review or to the PEDT questionnaire) increased the rate of patients classified in the “S” domain to 65.1% (400/614). This rate increased to 70.5% (433/614) and 70.7% (435/614) considering domains of IIEF-5 for orgasmic function and sexual desire and finally to 86.3% (530/614) also considering the reply to question 2b of NIH-CPSI.

Patients were classified according to three different versions of the UPOINTS system: UPOINTS-1 (rating S by erectile dysfunction alone), UPOINTS-2 (rating S by erectile dysfunction plus premature ejaculation), and UPOINTS 5 (rating S by all the five parameters of erectile dysfunction, premature ejaculation, orgasmic dysfunction, desire loss, and ejaculatory pain). Sexual dysfunction was rated at 49.8%, 65.1%, and 86.3%, respectively. The median number of positive domains was 4, 4, and 5, respectively.

The number of positive domains in UPOINTS-1 was significantly correlated with total NIH-CPSI score (0.250, *p* = 0.000), pain NIH-CPSI score (0.154, *p* = 0.018), micturition NIH-CPSI (0.273, *p* = 0.000) and QoL NIH-CPSI (0.232, *p* = 0.000).

Similarly, the number of positive domains in UPOINTS-2 and UPOINTS-5 were significantly correlated to total NIH-CPSI score and sub-scores for pain, micturition, and QoL (Table 3). The correlation coefficient r of Spearman between the number of positive domains of UPOINT and symptom severity slightly decreased when the “S” domain based on erectile function was added to UPOINT classification (UPOINTS-1) but increased progressively when “S” was based on more parameters of sexual dysfunction (UPOINTS-2 and UPOINTS-5).

UPOINTS-1 and UPOINTS-2 were negatively correlated with all the IIEF-15 scores, whereas UPOINTS-5 was correlated with IIEF-15 sub-scores for intercourse, orgasmic, desire, and satisfaction but not with IIEF-15 for erectile function.

### 3.7. A New Tool for Investigating Sexual Function in CBP

Finally, we simulated the effect of a new tool for investigating sexual dysfunction in patients with CBP. The first version included a 3-question inquiring about erectile function, premature ejaculation, and orgasmic function. Each question had a binary (Y/N) answer. The question on erectile function was derived from question 1 of IIEF (positive if score ≤ 2), that on PE from question 2 of PEDT (positive if score ≥ 3), and that on orgasmic function from question 10 of IIEF-15 (positive if score ≤ 2). In the second version, an ejaculatory pain question derived from question 2b of NIH-CPSI was added. The results of the two questionnaires were very well correlated with the scores of both NIH-CPSI and IIEF-15 (Table 4).

The 4-item questionnaire correlates positively with all chronic prostatitis symptom severity scores (NIH-CPSI), while the 3-question questionnaire is not correlated with the NIH-CPSI score for voiding symptoms; however, the correlation indices with sexual dysfunction domains of the 4-question score are lower than the 3-question one, so the simpler 3-question questionnaire may be preferred.

## 4. Discussion

A higher prevalence of erectile dysfunction and premature ejaculation in patients with moderate or severe CP/CPPS when compared to the general population has been confirmed in recent studies after adjustment for numerous epidemiological risk factors [15,16]. CP/CPPS would have a specific impact on erectile dysfunction in middle-aged men [17].

Some authors have emphasized the role of chronic pain, stress, and depression associated with CP/CPPS in the pathogenesis of sexual dysfunction in these patients [18], although data from other studies suggest that sexual dysfunction may occur independently of mental distress [19]. On the other hand, the impact of the disease on sexual dysfunction could be increased by specific personality traits [20].

A study evaluated the possible role of changes in hormone levels in the onset of erectile dysfunction in patients with CP/CPPS without demonstrating correlations with testosterone levels, while only prolactin levels were shown to be inversely correlated with the severity of erectile dysfunction [21].

In our series, we observed that age was an independent predictor of erectile dysfunction in CP/CPPS patients. This can be explained by the increasing prevalence with age of BPH [22] and several other pathologies related to sexual dysfunction, such as cardiovascular disease, diabetes, and depression [23].

Multivariate logistic regression analysis of data from a large population of male adult subjects from the NHANES 2001-2004 identified BPH as an independent risk factor for ED in the 60–80 years age group (OR = 1.93; 95% CI, 1.18–3.18, *p* = 0.02) [24]. The possible causes of this association are many, including alterations of nitric oxide (NO)-cyclic guanosine monophosphate (cGMP) pathway, enhanced RhoA-Rho-kinase (ROCK) contractile signaling, autonomic adrenergic hyperactivity, and pelvic atherosclerosis [22]. Unfortunately, there is a lack of recent studies evaluating the impact of genital tract infection on sexual dysfunction.

When we compared the effects on sexual function of infections diagnosed from cultures of different biological fluids from the genital tract (semen, EPS/VB3) we were not able to demonstrate any significant difference.

However, the demonstration of pathogens in a genital sample does not automatically mean that the infection originates from the organ from which that fluid comes (prostate or seminal vesicles) because the origin of the genital samples cannot be attributed exclusively to a specific organ. The anatomy of the genital tract is complex for sharing the terminal urinary tract in which ejaculatory ducts and the ducts of other male accessory glands are void. To obtain a sample of certain seminal origin, it is necessary to use invasive procedures such as transperineal needle sampling [25]. The prostate massage used in the Meares–Stamey test is an artifice to obtain a sample that is as representative as possible of the prostate fluid.

We cannot, therefore, consider the semen as exclusively representative of infection of the seminal vesicles and, vice versa, EPS/VB3 as a unique measure of the infection of the prostate tissue.

For these reasons, we cannot confirm previous observations that reported a higher frequency of sexual dysfunction (52%) in infertile patients with localized infection in male accessory glands according to transrectal and scrotal ultrasound evaluation [26,27].

Regarding the type of pathogens, Gram-negative infections, predominantly Enterobacterales, are more frequently associated with moderate to severe erectile dysfunction and orgasmic dysfunction, which occur less frequently in patients with infections from other pathogens.

Recent studies have emphasized the role of Enterobacterales in enhancing inflammatory response [28]. Microbe-associated molecular patterns (MAMPs) are molecules located on the bacterial surface that interact with the receptors on immune cells to trigger inflammation [29]. In particular, Enterobacterales possess a more immunostimulatory version of the endotoxin lipopolysaccharide (LPS), which interacts with toll-like receptor 4 (TLR-4) on immune cells. Enterobacterales also present unmethylated immunostimulatory activity through an interaction with TLR-9 [30].

Studies in patients with inflammatory bowel disease [31] reported the role of Enterobacterales in increasing the levels of IL-8, tumor necrosis factor (TNF)-α, and IL-1β.

In our series, erectile and orgasmic dysfunction is less affected by infections from atypical pathogens. This observation can be explained by the higher frequency of *Ureaplasma urealyticum* in comparison to *Chlamydia trachomatis* in the series (106 vs. 48).

Although *Ureaplasma urealyticum* has been associated with urethritis, it is a common opinion that asymptomatic carriage of these bacteria is more common [32], unlike *Chlamydia trachomatis,* which has been associated with a higher incidence of erectile dysfunction and premature ejaculation [33,34].

An interesting finding of this study was that while Gram-negative pathogens are associated with more acute sexual symptoms, Gram-positive strains are associated with a higher frequency of prostatic calcifications at ultrasound. These images can be associated with the presence of biofilms [35]. A study of a large CBP population showed that biofilm-producing bacteria are found in 85% of patients with CBP and that they are associated to a worse clinical response to antibiotic therapy [36].

The higher frequency of calcifications in Gram-positive aligns with other studies that have described the ability to produce biofilms by Gram-positive bacteria, particularly enterococci [37].

The rate of patients positive for each domain of the UPOINT system varies greatly depending on the evaluation criteria and the characteristics of the study population.

In previous studies, the rate of the urinary domain ranged from 63 to 76%, of the psychosocial 15.5–74%, of organ-specific 45–75.8%, of infection 3.4–34%, of neurologic/systemic 5.1–46%, and of tenderness 50–75.8% [38,39,40].

In our study, patients were assigned to the urinary domain (U) in 51.8%, psychologic domain (P) in 28.3%, organ-specific domain (O) in 76%, neurologic domain (N) in 41%, and tenderness (T) in 52.2%. All patients were positive for domain I (infection).

The number of positive UPOINT domains was correlated with NIH-CPSI score as previously demonstrated by other Authors [39,40].

The addition of an “S” domain for sexual dysfunction was evaluated contrastingly. In a multivariate analysis, Samplaski et al. [41] showed that adding a “Sexual Dysfunction” domain did not affect the relationship between the total number of positive UPOINT(S) domains and NIH-CPSI score. Arda et al. [39] found no correlation between ED severity and the number of positive UPOINT domains or NIH-CPSI scores. 

However, the assignment of the “S” phenotype according to the modified UPOINT system, depends on the choice of the parameters to use for assignment to the domain.

Using the IIEF score for the erectile domain (IIEF 1–5,15) the percentage of patients with sexual dysfunction in the present study was calculated 49.8%, which increased to 86.3% if the rate of PE and other domains of IIEF were considered. According to other Authors the rate of the subdomain of sexual dysfunction ranged from 39.9 to 62% [39,40].

A comprehensive evaluation of all aspects of sexual dysfunction shows that a partial component of sexual dysfunction is present in almost all patients with chronic bacterial prostatitis.

The scores of the answers to the different questionnaires that investigate the presence of the different sexual symptoms are not correlated with each other, indicating that each question investigates a specific aspect of the varied clinical manifestations of sexual dysfunction.

For this reason, there is a need for a simple questionnaire that can summarize in a few questions all aspects of sexual dysfunction associated with chronic bacterial prostatitis. The answers to 3 or 4 simple questions on the various aspects of sexual function produce a score that is highly correlated with the NIH-CPSI score for measuring symptoms of chronic prostatitis.

### Limitations

The first limitation of our study is the lack of a control group to confirm that symptoms of sexual dysfunction are more frequent in patients with CBP than in age-matched controls. However, this observation has already been confirmed by numerous studies and two meta-analyses. The rates of erectile dysfunction and premature ejaculation observed in our series (49.8% and 40.7%, respectively) [4,5,6] align with what was observed in previous meta-analyses (30–50% and 40%, respectively). Furthermore, in a previous study, we observed a higher rate of erectile dysfunction (39 vs. 33%) and ejaculatory disorders (63 vs. 52%) in patients with chronic bacterial prostatitis compared to those with non-bacterial prostatitis [1].

The study has some other limitations that, however, do not affect the results of our extensive analysis. The PEDT questionnaire was administered only to a subgroup of 111 patients, not allowing a precise evaluation of the presence and extent of premature ejaculation in all patients, who were assessed in most cases with an unstructured interview, which, however, proved to be efficient in highlighting the presence of this disorder.

The lack of a specific ultrasound evaluation to investigate the morphology of seminal vesicles did not allow us to demonstrate with certainty the involvement of vesicles in genital tract infection.

## 5. Conclusions

In conclusion, infections by Gram-negative are associated with more sexual morbidity in patients with CBP. Patients with infection by Gram-negative strains showed a higher rate of moderate to severe erectile dysfunction than patients with atypical pathogens, and patients with both Gram-positive and Gram-negative infections showed more frequent orgasmic dysfunction than patients with infection by atypicals.

The assignment of the “S” phenotype to the UPOINT system for the classification of CBP patients increases its efficacy in describing the severity of the disease, provided that the attribution of the “S” domain is made considering not only the erectile function but all the domains of sexual function.

Therefore, there is a need to use a specific questionnaire to assess sexual function in patients with chronic prostatitis that takes into account erectile function in combination with all aspects of sexual dysfunction, including premature ejaculation and orgasmic dysfunction. A brief questionnaire with three questions could be used in clinical practice, avoiding the time-consuming administration of several questionnaires.

## Figures and Tables

**Table 1:** Demographics and clinical characteristics of 614 patients with chronic bacterial prostatitis.

Demographic and Clinical Parameters		Questionnaire Scores	
Age (years)	45.6 ± 13.9	NIH-CPSI pain	10.4 ± 5.7
PSA (ng/mL)	2.3 ± 2.8	NIH-CPSI micturition	4.5 ± 2.7
Qmax (mL/s)	16.1 ± 9.3	NIH-CPSI QoL	7.8 ± 2.7
PVR (mL)	43.1 ± 55.9	NIH-CPSI total	22.7 ± 7.2
WBC VB3 (n° hpf)	9.1 ± 10.8	IPSS	11.9 ± 7.4
WBC semen (n° hpf)	9.1 ± 8.1	IIEF1–5,15 erectile function	21.9 ± 7.8
Prostatic calcification	255/614 (41.5%)	IIEF6–8 intercourse	9.8 ± 4.0
Hematospermia	183/614 (29.8%)	IIEF 9–10 orgasmic function	7.2 ± 2.6
Premature ejaculation	250/614 (40.7%)	IIEF 11–12 desire	6.8 ± 2.5
Risk factors for erectile dysfunction	37/614 (6%)	IIEF 13–14 overall	6.5 ± 2.5
		PEDT Total *	7.027 ± 5.290

* PEDT in 111 patients.

**Table 2:** Erectile function, orgasmic function, sexual desire, premature ejaculation, and hemospermia in patients with pathogens isolated in different biological samples and in patients with different types of pathogens.

		EPS/VB3	Semen	EPS/VB3 + Semen	Sig
Patients	N°	155	307	141	
Erectile function IIEF 1–5,15	≤16 N° (%)	36 23.2%	53 17.2%	33 23.4%	0.206
Orgasmic function IIEF 9–10	≤2 N°	41	89	39	0.609
Impaired desire IIEF 11–12	≤2 N°	45	84	36	0.646
Premature ejaculation	Yes N°	50	144	50	0.172
Hemospermia	Yes N° (%)	39/150 26%	93/329 28.3%	48/118 40.7%	0.241
Prostatic calcifications	Yes N° (%)	65/150 43.3%	127/329 38.6%	60/118 50.8%	0.971
**EPS/VB3**		Gram-neg	Gram-pos	Atypical	Sig
Patients	N°	91	64	47	
Erectile function IIEF 1–5,15	≤16	40 43.9%	20 31.2%	9 19.1%	0.045
Orgasmic function IIEF 9–10	≤2	43 47.2%	28 43.7%	9 19.1%	0.004
Impaired desire IIEF 11–12	≤2	44/116	23/99	14/53	0.077
Premature ejaculation	yes	42/116	37/99	21/53	0.913
Hematospermia	yes	46/116	29/99	12/53	0.190
Prostatic calcifications	yes	45/116	53/99	27/53	0.108
**Semen**		163	176	109	
Erectile function IIEF 1–5,15	≤16	32/142	29/196	25/107	0.117
Orgasmic function IIEF 9–10	≤2	40/143	56/196	32/108	0.955
Impaired desire IIEF 11–12	≤2	40/143	44/196	36/108	0.275
Premature ejaculation	yes	59/143	87/196	48/108	0.137
Hematospermia	yes	51 31.2%	67 38.0%	23 21.1%	0.011
Prostatic calcifications	yes	53 32.5%	89 50.5%	45 41.2%	0.003

**Table 3:** Correlation between the number of positive domains of UPOINT, UPOINTS-1, UPOINTS-2, and UPOINTS-5 and the scores of NIH-CPSI (total, pain, micturition, QoL) and IIEF-15 (erectile, intercourse, orgasmic, desire, intercourse, satisfaction). UPOINT reported the assessment of five domains related to the clinical presentation of CP/CPPS: urinary, psychologic, organ-specific, neurologic, and tenderness. UPOINTS added an “S” (sexual) domain, which was assessed as positive by the presence of erectile dysfunction (UPOINTS-1) or by the presence of both erectile dysfunction and/or premature ejaculation (UPOINTS-2) or by the presence of at least one of the five main sexual symptoms (UPOINTS-5). An increase in the Spearman correlation coefficient of the number of positive domains for each patient with NIH-CPSI points demonstrates that the addition of the “S” domain improves the reporting of CP/CPPS severity.

		NIH-CPSI Total	NIH-CPSI Pain	NIH-CPSI Mict	NIH-CPSI QoL	
UPOINT	*r*	0.262	0.164	0.272	0.245	
	*p*	0.000	0.000	0.000	0.000	
UPOINTS-1	*r*	0.250	0.154	0.273	0.232	
	*p*	0.000	0.018	0.000	0.000	
UPOINTS-2	*r*	0.268	0.176	0.266	0.247	
	*p*	0.000	0.009	0.000	0.000	
UPOINTS-5	*r*	0.288	0.194	0.284	0.252	
	*p*	0.000	0.004	0.000	0.000	
		IIEF-15 erectile	intercourse	orgasmic	desire	satisfaction
UPOINT	*r*	−0.006	−0.027	−0.025	−0.044	−0.050
	*p*	0.876	0.488	0.536	0.280	0.245
UPOINTS-1	*r*	−0.211	−0.171	−0.161	−0.183	−0.197
	*p*	0.000	0.000	0.000	0.000	0.000
UPOINTS-2	*r*	−0.138	−0.118	−0.116	−0.127	−0.143
	*p*	0.001	0.004	0.004	0.002	0.001
UPOINTS-5	*r*	−0.077	−0.081	−0.086	−0.095	−0.106
	*p*	0.057	0.046	0.034	0.019	0.014

**Table 4:** Correlation between the values of a new score with values of NIH-CPSI and IIEF-15 scores. The 3-questions score was based on the replies to three questions on erectile dysfunction, premature ejaculation, and orgasmic dysfunction. The 4-question score included a fourth question on ejaculatory pain. Both versions performed well in predicting scores of symptoms of prostatitis, although the 4-question score achieved a better correlation with all the domains explored by questionnaires.

		NIH-CPSI Total	NIH-CPSI Pain	NIH-CPSI Mict	NIH-CPSI QoL	
3-questions	*r*	0.177	0.168	0.063	0.186	
	*p*	0.000	0.000	0.119	0.000	
4-questions	*r*	0.307	0.321	0.123	0.275	
	*p*	0.000	0.000	0.002	0.000	
		IIEF-15 erectile	intercourse	orgasmic	desire	satisfaction
3-questions	*r*	−0.408	−0.368	−0.490	−0.377	−0.400
	*p*	0.000	0.000	0.000	0.000	0.000
4-questions	*r*	−0.355	−0.301	−0.445	−0.311	−0.351
	*p*	0.000	0.000	0.000	0.000	0.000

## Data Availability

The raw data supporting the conclusions of this article will be made available by the authors on request.

## Abbreviations

The following abbreviations are used in this manuscript:

CBP	chronic bacterial prostatitis
ED	erectile dysfunction
PE	premature ejaculation
EPS	expressed prostatic secretion
VB3	voiding bladder 3
PVR	post-voiding residual
NIH-CPSI	National Institute of Health-Chronic Prostatitis Symptom Index
IPSS	International Prostatic Symptom Score
IIEF-15	International Index of Erectile Function-15
UPOINT	Urinary Psychosocial Organ-specific Infection Neurologic Tenderness
